# Prion Protein Misfolding, Strains, and Neurotoxicity: An Update from Studies on Mammalian Prions

**DOI:** 10.1155/2013/910314

**Published:** 2013-12-24

**Authors:** Ilaria Poggiolini, Daniela Saverioni, Piero Parchi

**Affiliations:** ^1^Dipartimento di Scienze Biomediche e Neuromotorie (DiBiNeM), Università di Bologna, 40123 Bologna, Italy; ^2^IRCCS Istituto delle Scienze Neurologiche, Via Altura 3, 40139 Bologna, Italy

## Abstract

Prion diseases, also known as transmissible spongiform encephalopathies (TSEs), are a group of fatal neurodegenerative disorders affecting humans and other mammalian species. The central event in TSE pathogenesis is the conformational conversion of the cellular prion protein, PrP^C^, into the aggregate, **β**-sheet rich, amyloidogenic form, PrP^Sc^. Increasing evidence indicates that distinct PrP^Sc^ conformers, forming distinct ordered aggregates, can encipher the phenotypic TSE variants related to prion strains. Prion strains are TSE isolates that, after inoculation into syngenic hosts, cause disease with distinct characteristics, such as incubation period, pattern of PrP^Sc^ distribution, and regional severity of histopathological changes in the brain. In analogy with other amyloid forming proteins, PrP^Sc^ toxicity is thought to derive from the existence of various intermediate structures prior to the amyloid fiber formation and/or their specific interaction with membranes. The latter appears particularly relevant for the pathogenesis of TSEs associated with GPI-anchored PrP^Sc^, which involves major cellular membrane distortions in neurons. In this review, we update the current knowledge on the molecular mechanisms underlying three fundamental aspects of the basic biology of prions such as the putative mechanism of prion protein conversion to the pathogenic form PrP^Sc^ and its propagation, the molecular basis of prion strains, and the mechanism of induced neurotoxicity by PrP^Sc^ aggregates.

## 1. Introduction

Prion diseases, also known as transmissible spongiform encephalopathies (TSEs), are rapidly progressive neurodegenerative disorders that affect many species of mammals. In humans, they comprise Creutzfeldt-Jakob disease (CJD), fatal familial insomnia (FFI), kuru, Gerstmann-Sträussler-Scheinker disease (GSS), and the recently described variably protease-sensitive prionopathy (VPSPr), whereas natural TSEs in animals include scrapie of sheep and goats, bovine spongiform encephalopathy (BSE), and chronic wasting disease (CWD) in deer and elk.

Prion diseases belong to the growing group of disorders that are attributed to misfolding and ordered aggregation of proteins, which include Alzheimer's disease, Parkinson's disease, systemic amyloidosis, and many others. In prion disease, in particular, the cellular prion protein, PrP^C^, after partial misfolding, converts into a partially protease-resistant disease-associated isoform, PrP^Sc^, which aggregates in the brain and forms deposits that are associated with the neurodegenerative changes.

Distinguishing features of prion diseases among these disorders, however, are their wide phenotypic spectrum, the multiple apparent ethiologies (e.g., sporadic, genetic, and acquired), and the transmissibility between individuals, a characteristic which has allowed the early development of experimental models. This has led to the important discovery that mammalian prions occur, like conventional infectious agents, in a variety of different strains: these are defined as natural isolates of infectious prions characterized by distinctive clinical and neuropathological features, which are faithfully recapitulated upon serial passage within the same host genotype. The different strains of the TSE agent or prion are believed to be the main cause of TSE phenotypic diversity. In addition, the host variability in the gene encoding PrP^C^ (*PRNP*), as determined by polymorphisms or mutations, also modulates the disease phenotype. In this review, we focus on three fundamental aspects of the basic biology of prions, which, despite the significant recent advances, remain unsolved. They include the molecular mechanisms of PrP^C^ to PrP^Sc^ conversion, the role of PrP^Sc^ in strain determination, and the mechanism of PrP^Sc^ aggregate-induced neurotoxicity. Due to the space constraint and the main expertise of the authors, emphasis is given to evidence obtained from the study of naturally occurring diseases, particularly in humans, and from animal models.

## 2. **PrP^C^-PrP^Sc^** Conversion

### 2.1. Structural Changes Associated with PrP^C^ to PrP^Sc^ Conversion

Understanding the structural features of PrP^Sc^ remains a key issue to gain the ultimate insight into the molecular basis of prion formation and propagation. Unfortunately, the insoluble nature of PrP^Sc^ has hampered most efforts to determine its structure by preventing the use of high-resolution techniques such as NMR or X-ray crystallography. Therefore, only partial structural information is available from low-resolution approaches such as Fourier transform infrared spectroscopy (FTIR), electron microscopy (EM), immunoassays, fiber X-ray diffraction, and limited proteolysis [1–9]. Full-length PrP^C^ encompasses a poorly definite domain at the N-terminal end of the protein (which spans ~100 residues), a globular domain in the central portion (residues 125–228), and a short flexible C-terminal domain, ending with the GPI anchor (residues 229-230/231) [10]. The globular domain is composed of three *α*-helices and two antiparallel *β*-sheets, separated by short loops and kept together in their final tertiary structure by interactions between the exposed amino acidic lateral chains that are in close contact with each other when the protein is correctly folded [10]. The conversion of PrP^C^ into the pathological conformer PrP^Sc^ is characterized by a significant increase of *β*-sheet secondary structure. Indeed, FTIR and circular dichroism (CD) spectroscopy experiments indicate a dramatic difference in the secondary structure between the two isoforms. While PrP^C^ contains 47% *α*-helix and 3% *β*-structure, PrP^Sc^ holds 17–30% *α*-helix and 43–54% extended *β*-structure, the range being partially due to the multiple forms and lengths of PrP^Sc^ [2, 11].

Taking advantage of the available low-resolution structural information and constraints about PrP^Sc^ and of computational techniques, different theoretical models have been proposed to describe the putative PrP^Sc^ structure. The *β*-helical model is based on fiber X-ray diffraction and computer modeling techniques and proposes that the segment ~90–175 forms a four-stranded *β*-sheet core organized in a *β*-helical configuration, whereas helices *α*2 and *α*3 would retain their native conformation [3]. An alternative “spiral” model is based on molecular dynamics simulations and indicates that during PrP^C^ conversion a longer single *β*-strand is generated from the elongation of the two native *β*-sheets. The newly formed *β*-strand would interact with other PrP molecules and, in turn, lead to polymerization [12]. In both models the basic subunit of the oligomers is considered a trimer. According to the authors who have proposed the spiral model, however, the *β*-helical model is in disagreement with several critical constraints: notably, it would not fit within the unit cell packing dimensions of the EM data for which it was modeled and would be inconsistent with antibody mapping studies, enzyme cleavage sites, and fibril disaggregation profiles [12]. Furthermore, the results of recent deuterium exchange experiments on brain-derived PrP^Sc^ showed that the region from residue ~90 to the entire C-terminus displays slow exchange rates that are typical for a structure consisting of a continuum of *β*-strands [13]. These findings from Surewicz's group appear inconsistent with both the “*β*-helical” and the “spiral” models, which are assuming an incomplete conversion of the *α*-helical structures into *β*-sheet and add further controversy to the issue. Of course, current models do not rule out the possibility that there are other structures that would satisfy the experimental constraints. Indeed, given that in mammals more than a dozen of different prion strains are documented, a higher structural heterogeneity is expected and should be explained.

### 2.2. Effects of *PRNP* Mutations

Several mutations in the PrP gene (*PRNP*) account for the genetic or familial form of human prion disease, in which the conversion of PrP^C^ into PrP^Sc^ is thought to occur spontaneously, triggered by the mutation. About forty mutations linked to familial CJD, GSS, FFI, or other atypical phenotypes have been identified to date [15]; they have been linked to a plethora of effects at both structural and clinicopathological levels. Based on their position in the gene, their effect, and the type of residue replaced, *PRNP* mutations can be classified in several groups: N-terminal or C-terminal mutations, missense, insert, or STOP-codon mutations, salt bridge-affecting, polar mutations, and hydrophobic or GPI-signal-peptide mutations [16].

Based on *in vitro* studies it has been proposed that disease-linked mutations increase the likelihood of PrP^C^ misfolding by thermodynamically destabilizing the protein [17–20]. However, this cannot be taken as a general mechanism because individual mutations differently (or barely) affect PrP^C^ stability. Besides influencing the stability of PrP^C^, mutations may also alter its surface properties, thus triggering an abnormal interaction with other not yet identified cofactors, or causing an aberrant trafficking and accumulation inside the cell [16].

Atomic structural details, obtained using solution-state NMR spectroscopy, are available only for a few pathological human (Hu) PrP mutants. Based on the structural comparison of the folded domain (residues 125 to 228) of HuPrP carrying the CJD-linked E200K or V210I [21] mutations and the GSS-linked Q212P [22] mutation, it has been proposed that pathological mutants affects the aromatic and hydrophobic interactions between residues clustered at the interface of the *β*2-*α*2 loop and the C-terminal half of the *α*3 helix. The disruption of these interactions and the consequent exposure to the solvent of the hydrophobic core may represent a common effect of the three mutants, which has led to the proposal that the early stage of prion conversion possibly involves the critical epitope formed by the *β*2-*α*2 loop and the *α*3 helix. Similar findings have been obtained with the X-ray crystal structure of both F198S and D178N mutants [23] and molecular dynamics experiments [24, 25]. 

HuPrP pathological mutants were also explored in several murine models. In particular, various transgenic (Tg) mouse models overexpressing mutated PrP constructs (or wild-type PrP) were developed in order to determine whether PrP is *per se* sufficient to give rise to disease and generate infectivity. In an early controversial study Hsiao and colleagues reported that Tg mice overexpressing the mutated PrP P101L, an homologous of the P102L substitution associated with the GSS syndrome in humans, spontaneously develop a clinical-pathological phenotype which propagated disease in inoculated Tg 196 mice expressing lower levels of mutant protein, suggesting that pathogenic PrP gene mutations resulted in the spontaneous formation of PrP^Sc^ and *de novo* production of prions [26]. Subsequent studies, however, have shown that the Tg 196 mice also spontaneously develop the disease in late life as a consequence of PrP overexpression, making the apparent prion propagation observed in this model more accurately characterized as disease acceleration rather than transmission [27]. Remarkably, disease transmission of brain extracts from Tg animals overexpressing the P101L mutation neither occurred to wild-type nor to Tg mice expressing MoPrP-P101L from two transgene copies that do not develop disease spontaneously in their natural lifespan [27], which is in full agreement with a previous study from Manson's group showing that *PRNP* gene-targeted 101LL mice expressing MoPrPP101L failed to develop the neurodegenerative disease spontaneously [28].

In line with the concept expressed above, several subsequent studies reported that Tg mice overexpressing PrP mutants often develop neuropathological features reminiscent of human TSEs, although in most cases the inoculation of their brain extracts in wild-type animals neither reproduced the main feature of the disease nor generated infectivity [29–34].

Results contradicting this general observation, however, have also been reported. Transgenic mice moderately overexpressing a mutant mouse PrP carrying two point mutations (170N and 174T) that are found as normal variants in the rigid loop of elk PrP spontaneously develop spongiform encephalopathy and PrP plaque deposition in the brain [35]. Repeated subpassages in Tg20 mice showed transmission of disease to wild-type mice and propagation of protease-resistant PrP^Sc^. Similarly, Lindquist and collaborators were able to generate knock-in mice expressing the mouse equivalent of the PrP mutation (i.e., D178N-M129) associated with FFI. These mice developed *de novo* prion diseases with neuropathological traits similar to FFI that was transmissible to wild-type mice carrying the same 3F4 epitope [36]. A very similar result has been recently obtained by the same group using knock-in mice carrying the mouse equivalent of the most common human mutation (i.e., E200K) associated with genetic CJD. These mice developed the hallmark features of CJD, namely, spongiosis and proteinase K (PK)-resistant PrP aggregates. Furthermore, brain extracts from these mice caused a transmissible neurodegenerative disease after intracerebral inoculation in WT mice [37]. Finally, infectious prions were also reported to form spontaneously, even before the onset of the clinical symptoms, in chimeric mouse/human transgenic mice (called TgMHu2M), also expressing the CJD-linked E200K mutation [38]. Thus, according to these four studies, the introduction of a single (or two) amino acidic change(s) in *PRNP* in a critical position can cause remarkably different neurodegenerative diseases and may be sufficient to create distinct protein-based infectious prions. 

Tg mice lines expressing human *PRNP* mutations were also used to study the effect of the mutation on disease susceptibility. Transgenic mice carrying the P101L mutation in PrP had remarkable differences in incubation time compared with wild-type littermates, following inoculation with several prion strains from human, hamster, sheep, and murine sources, suggesting a critical role for the structurally “flexible” region of PrP in agent replication [30]. In another study, Asante and collaborators [29] studied mouse lines homozygous for the human PrP102L, 129 M or for human PrP200 K, 129 M transgenes both expressed on *PRNP* null background. Although both lines did not develop spontaneous neurodegeneration, they showed a different susceptibility to inherited prion diseases. While PrP102L, 129 M were permissive to homotypic P102L prions and not to sCJD prions, PrP200 K, 129 M showed a similar susceptibility to both the E200K *inoculum* and classical sCJD prions [29]. Tg mice lines carrying *PRNP* mutations have also been used to unveil molecular pathways that are activated by the expression of mutant PrP, which may lead to neuronal dysfunction. In a recent study Senatore et al. shed light on the effects of insertional mutants on synaptic transmission [39]. Using Tg mice expressing a PrP insertional mutation linked to familial prion disease [31], they pinpointed the existing relationship between the early motor behavioral abnormalities and the impaired glutamatergic neurotransmission in cerebellar granule neurons. In particular, they showed that the misfolded mutant PrP undergoes an aberrant intracellular trafficking causing the intracellular accumulation of the voltage gated calcium channel *α*2*δ*-1 subunit, which results in the disruption of the cerebellar glutamatergic neurotransmission [39].

### 2.3. Cellular Cofactors Featuring in PrP^C^ Conversion and Prion Propagation

Several lines of evidence suggest that different classes of cofactors, possibly acting as chaperones, can influence PrP^C^ conversion and prion propagation [40, 41]. To date, two types of cofactors, lipids and polyanions, have been implicated (Table 1), although their precise mechanism of action remains unclear. Among linear polyanions, glycosaminoglycans (GAGs) and sulfated polysaccharides such as pentosan polysulfate or heparan sulfate were shown to influence prion conversion *in vitro* [42–45] (Table 1) possibly by facilitating the formation of PrP^C^-PrP^Sc^ complexes through multiple simultaneous interactions with several PrP molecules [42].

Most significantly, host-encoded RNA was shown to facilitate the prion-seeded conversion of PrP^C^ to PrP^Sc^ *in vitro* [46–50]. However, whether RNA acts as a mere catalyst of the PrP misfolding process or, alternatively, is associated with the infectious particle and contribute to determine the prion strain specificity is still unsolved. A recent study showed that the requirement of RNA for *in vitro *amplification of PrP^Sc^ is species dependent, with only hamster-derived PrP^Sc^ being largely dependent on the presence of RNA, whereas mouse-derived PrP^Sc^ is not [51]. Another study showed similar RNA-dependent amplifications of six hamster prion strains [52]. DNA and phospholipids have also been implicated as cofactors modulating prion replication *in vitro*. The polymerization of the mouse recombinant PrP (rPrP) was enhanced in presence of nucleic acids and sequence-specific DNA binding to rPrP converted it from a *α*-helical conformation to a soluble, *β*-sheet enriched isoform similar to that found in the fibrillar PrP^Sc^ state [53, 54]. Unlike RNA, the essential membrane phospholipid phosphatidylethanolamine (PE) was described as a highly promiscuous cofactor that can promote prion propagation using rPrP molecules from different mammalian species [55]. Critical questions, which are still far from being fully answered, concerns the role, if any, of cofactors in modulating prion infectivity and the specific properties of prion strains. Preliminary data on *in vitro* reconstitute prions seem to indicate that the presence of cofactors enhances *in vivo* prion infectivity, whereas the data collected to date on the issue of strains appear to be inconsistent. For example, while the use of PE as unique cofactor in the propagation process allowed the adaption of two different native prion strains into the same unique output strain, suggesting that a single cofactor is able to force the conversion of different strains into a single strain having its own phenotypic features [56], in another study it was found that replication under RNA-depleted conditions does not modify RML prion strain properties [57].

### 2.4. Cellular Sites of PrP^Sc^ Formation

Being PrP^C^ a GPI-anchored protein, it mainly localizes in lipid rafts of cellular membranes where it can interact *in trans *with a variety of signaling molecules, including caveolin-1, Fyn, and Src tyrosine kinases [58], or with other cell-surface proteins as NCAM [59], stress-inducible protein 1 [60–62], vitronectin, lipoprotein receptor-related protein 1 [63, 64], or reelin [65].

Several lines of evidence suggest that lipid rafts are critically involved in the conversion of PrP^C^ into the pathological form PrP^Sc^. Using immortalized neuroblastoma cells ScN2a, chronically infected by the Rocky Mountain Laboratory (RML) prion strain, Naslavsky et al. showed that PrP^Sc^ is attached to lipid rafts [66] and that the amount of the abnormal protein inversely correlates with sphingomyelin levels [67]. Furthermore, using thin-layer chromatography and mass spectrometry, it has been found that the insoluble aggregates of N-terminally truncated PrP^Sc^ (i.e., PrP 27–30) contain small amounts of two host sphingolipids, galactosylceramide and sphingomyelin [68], which also supports the localization of PrP^Sc^ in rafts. Other data pointing to a raft-mediated conversion include the observations that depletion of cellular cholesterol or the replacement of PrP^C^ GPI-anchor with the transmembrane and cytosolic domain from nonrafts proteins diminished or prevented the formation of PrP^Sc^ [58]. More recent studies, however, highlighted the possibility that lipid rafts favour the conversion by bringing together PrP^Sc^ and PrP^C^, rather than by triggering PrP^C^ refolding [69]. Indeed, given their role in PrP^C^ folding and stabilization of its conformation, lipid rafts may even prevent PrP^C^ transconformation. According to this view the conversion would occur only after PrP^C^ exits in these domains. Finally, other studies also suggested that lipid rafts do not provide the environment in which PrP^C^-PrP^Sc^ refolding occurs, but rather promote PrP^Sc^ aggregation and fibrillization once the pathogenic misfolded protein has been produced elsewhere (reviewed in [70]).

Concerning the precise cellular site of conversion of PrP^C^ to PrP^Sc^, early studies pointed to the cell surface [71], which appears a plausible location particularly for the case of transmitted prion diseases, or to the endocytic pathway [72–75]. Subsequent studies further underlined the potential role in the conversion process of intracellular compartments such as the endosomal or lysosomal pathways, or even the ER [76–79]. Evidence for the conversion of PrP^C^ to PrP^Sc^ occurring shortly after internalization, during an endocytic process, is indeed numerous. After treatment of both scrapie-infected Syrian hamster brain and ScN2a cell lines with guanidine-hydrochloride, which allows epitope unmasking in native PrP^Sc^, the abnormal protein was primarily described intracellularly [75], where it was found to accumulate in lysosomes. In another study, using cryo-immunogold electron microscopy, PrP^Sc^ was found to be concentrated in early/recycling endosomes of neuritis of prion infected hippocampal neurons [77, 79]. Similarly, in three different neuronal cell lines infected with different prion strains more than 25% of PrP^Sc^ has been observed to colocalize with a marker for the early recycling compartment. Classic studies have also shown that PrP^Sc^ accumulates intracellularly as an N-terminal truncated form, which is generated after proteolytic cleavage in both endosomes and lysosomes [73, 74]. Supporting evidence for the role of endosomes or lysosomes in PrP^C^ conversion is also provided by the observations that an acidic pH triggers the conformational change of PrP^C^ to a PrP^Sc^-like form and that the lowering of the temperature to 18°C, supposedly by slowing the rate of PrP^C^ endocytosis, reduces PrP^Sc^ formation (reviewed in [80]).

Finally, the main cellular site of PrP^C^ and PrP^Sc^ location was also found to differ depending on the investigated cell line. In ScN2a cells, for example, PrP^C^ and PrP^Sc^ colocalize in the late-endosomial compartments, whereas in scrapie-infected hypothalamic (GT1—7) cells PrP^Sc^ is present in an additional vesicular compartment which is flotillin-1-positive [81].

As a whole, the data collected indicate that in most infected cell lines the conversion event occurs either on the cell surface or along the endocytic pathway, with PrP^Sc^ ultimately mainly accumulating in lysosomes. Nevertheless, other cellular sites might be also involved depending on the cell type, the prion strain, or the disease etiology.

## 3. **PrP^Sc^** and the Strain Phenomenon

The first demonstration of prion strains was obtained after transmission of distinct scrapie isolates [82]. When these sheep brain extracts were passaged to goats, a drowsy syndrome developed in some animals, while others had a scratching syndrome. A variety of scrapie strains were subsequently identified after passage through inbred mouse lines [83]. Properties that differentiate the strains are the length of incubation time following inoculation, the type and distribution of lesions (neuropathologic profile), and the pattern of intracerebral deposition of PrP^Sc^ [83–85]. The wide variety of scrapie strains has been traditionally seen as the major challenge to the protein only hypothesis [86, 87]. While in classical infectious diseases different strains of the agent are associated with variations in their nucleic acid genomes, the prion hypothesis implicates that PrP^Sc^ itself would encode the phenotypic properties of the strains.

Kascsak et al. [88] originally documented that the relative proportion of PrP^Sc^ glycoforms, the so called “glycoform ratio,” was associated with strain variability and could be used to differentiate strains of the scrapie agent when isolated in inbred mice. At about the same time, mouse strains ME7 and 139A scrapie associated fibrils (SAF) were shown to differ from hamster strain 263K SAF in terms of morphology, sedimentation rate, and sensitivity to PK digestion [89]. Noteworthy, these distinctive PrP^Sc^ physicochemical properties were initially considered an effect of the scrapie agent on PrP rather than an evidence for a role of PrP^Sc^ itself in strain determination. Indeed, the idea that the molecular basis of strain variation may lie in the structure of PrP^Sc^, as predicted by the prion hypothesis, was fully embraced only after Bessen and Marsh found that two strains of transmissible mink encephalopathy (TME), transmitted to inbred Syrian hamsters, give rise to PrP^Sc^ molecules with distinct electrophoretic mobility and degree of resistance to protease digestion [90]. The two TME strain-specific PrP^Sc^ have been subsequently propagated *in vitro* through nongenetic mechanisms [91], which has further strengthened the view that the self-propagation of distinct PrP^Sc^ conformers may represent the basis of the prion strain phenomenon.

Experiments of FFI transmission to Tg mice gave additional support to the idea that the diversity of prion strains is enciphered in the PrP^Sc^ structure [92]. Brain homogenates from subjects affected by FFI, which contained a PrP^Sc^ fragment after PK digestion (PrP^res^) of 19 kDa, and from subjects with sporadic CJD (sCJD) or a genetic CJD (gCJD) subtype linked to the E200K-129M haplotype (CJDE200K-129M), which contained a PrP^res^ fragment with a relative molecular mass of 21 kDa, were inoculated to syngenic mice. The endogenous PrP^res^ recovered in the affected animals consistently and precisely replicated the size of the corresponding human PrP^res^.

In 1998 Safar et al. [93] introduced the conformation-dependent immunoassay (CDI), which measures the extent of epitope exposure after GndHCl denaturation and is therefore assumed to measure indirectly the relative percent of PrP^Sc^ *β*-sheet and *α*-helical content. Eight mouse-passaged scrapie strains were analyzed for strain-specific differences in secondary structure [93]. By plotting the ratio of antibody binding to the denatured/native proteins as a function of the concentration of PrP^Sc^, the authors observed that each strain occupies a unique position, suggesting a distinct conformation.

FTIR spectroscopy has also been used to measure the secondary structure of both PK-treated and full-length PrP^Sc^. Caughey and colleagues have originally compared the conformations of PrP^Sc^ in the HY, DY, and 263K hamster TSE strains and found striking differences in their secondary structures [1]. Similarly, another team [94, 95] has subsequently found strain-specific differences in secondary structure, temperature stability, and hydrogen-deuterium exchange characteristics between purified PrP^Sc^ preparations obtained from three scrapie strains and the classical BSE strain after passage in hamster.

More recently, the issue of the relationship between PrP^Sc^ conformational stability and strain-specific properties, such as incubation time and *in vitro* replication efficiency, has been addressed. In 2006 Legname et al. [96] reported that a reduced resistance to GndHCl denaturation, indicative of a reduced conformational stability, correlates with a shorter incubation time in mouse adapted prion strains. Similarly, the stability of PrP^Sc^ aggregates both in terms of resistance to GndHCL induced denaturation and thermostability was inversely correlated with the capacity to induce a rapidly lethal disease [97]. The provided explanation for these observations is that a decrease of PrP^Sc^ stability increases PrP^Sc^ aggregate fragmentation resulting in an increase in agent replication that produces a correspondingly shorter incubation period and a more aggressive disease. The relationship between the stability of PrP^Sc^ aggregates and PrP^Sc^ replication investigated *in vitro* using the protein misfolding cyclic amplification (PMCA) paradigm [52] also supports a link between PrP^Sc^ conformational stability and fragmentation rate of PrP^Sc^ aggregates. Other data, however, suggest a more complex picture, especially *in vivo*, where additional factors, related to cellular processing, may also play a significant role. In apparent contrast with what was observed in mice, Ayers et al. [98] found that hamster-adapted scrapie strains with a short incubation period were more efficiently replicated, had a more stable conformation, and were more resistant to clearance from the soma of neurons than those with a longer incubation time which, in contrast, predominantly accumulated in glial cells. These results suggest that the progression of prion disease is also influenced by the balance between replication and clearance of PrP^Sc^ in neurons.

A potential new perspective to the study of PrP^Sc^ properties and their relationship to prion strains was opened by the characterization of the so-called “sensitive PrP^Sc^” (sPrP^Sc^), an isoform of abnormal PrP which is fully degraded at a PK activity comparable to that necessary to digest PrP^C^, despite maintaining other properties that are specific for PrP^Sc^ [99–101]. Evidence for sPrP^Sc^ being a biologically relevant species originally came from the study of PrP^Sc^ properties in naturally occurring prion diseases. Indeed, a fully PK-sensitive PrP^Sc^ has been detected in various phenotypically atypical variants of both human and animal prion diseases [102–107]. Furthermore, according to some studies [100, 108], sPrP^Sc^ represents an invariable and quantitatively significant component of prions, contributing up to 90% of the whole PrP^Sc^ signal even in classic TSEs such as sCJD and classical scrapie. Recent studies have also found a correlation between the relative amount of sPrP^Sc^ with strain-specific properties such as the incubation period after inoculation or the clinical duration of the disease [109, 110]. We also recently looked for sPrP^Sc^ in purified detergent-insoluble PrP^Sc^ sCJD preparations [111]. At variance with the findings above, however, our results showed that, irrespectively of the human prion strain, this slowly sedimenting sPrP^Sc^ represents a relatively minor component of abnormal PrP not exceeding 10% of total detergent-insoluble PrP^Sc^. Thus, this significant discrepancy, which may depend at least partially on methodological aspects or data interpretation [111], needs to be further explored and explained.

Although not essential for prion propagation [112], PrP glycosylation of asparagine residues at positions 181 and 197 represents another factor likely contributing to the diversity of mammalian prions. Indeed, differences in ratios of di-, mono-, and unglycosylated PrP^Sc^ have been detected among phenotypic subtypes of both human and animal TSEs and are commonly used to differentiate specific strains [113–116]. This is consistent with the notion that glycosylation is critical in determining and maintaining conformation and interaction of glycoproteins [117, 118]. However, it is at present unclear whether glycans affect the backbone conformation of PrP^Sc^ molecules or rather modulate the interaction of these molecules by introducing specific steric constraints or by forming crucial intermolecular contact sites between PrP^Sc^ monomers [119]. In a recent elegant study Cancellotti et al. [120] have demonstrated that the passage in Tg mice expressing a PrP partially or completely lacking the N-glycan moieties affected the phenotypic characteristics of at least one TSE agent strain. Given that these changes could be successfully retained on passage in wild-type mice, it has been concluded that infectious properties of a TSE strain can be altered by posttranslational changes to host PrP, possibly as the result of the selection of mutant TSE strain.

Taken together all these pieces of evidence provide strong support to the argument that different PrP^Sc^ conformers encipher the prion “strains.” Nevertheless the direct proof for this contention is not yet available. Until a higher resolution picture of PrP^Sc^ provides the precise molecular-level details surrounding the puzzling phenomenon of prion strains and the conformational adaptability of PrP observed upon cross-species transmission, questions and alternative interpretations of the data will remain. For example, we cannot yet be sure of whether the distinctive properties of PrP^Sc^ directly reflect the tertiary conformation of monomers or are determined by interactions between PrP^Sc^ and other molecules acting as cofactors. PrP^Sc^ is extracted from the brain in a highly aggregated state and the heterogeneity in size of the PK digested protein core may well reflect the quaternary rather than the tertiary structure of the molecule. Similarly, the extent of conversion of each glycoform of PrP^Sc^, which ultimately determines the glycoform ratio of PrP^res^, may also represent a signature imparted by another molecule that interacts with PrP. Finally, the central question that still remains to be answered is how an identical primary sequence can drive different tertiary conformations in the prion protein, if no other informational molecule exist. Even more difficult to explain in terms of PrP^Sc^ structural plasticity are other two fundamental aspects of the biology of prions, the so-called “species barrier,” that is, the phenomenon for which a strain must adapt to a new species host with a typical delay in incubation time, or even the loss of infection ability in that species, and, above all, the fact that prion strains, like conventional infectious agent strains, incur in spontaneous “mutations.” The latter phenomenon is often explained with the quasispecies hypothesis [121], which predicts that PrP^Sc^ with different conformations may be present at low levels in an infectious inoculum and that the variant most suitable for replication in a particular host is selected to become the dominant component of the population [122, 123]. However, evidence for large numbers of conformations is still lacking nor is it clear whether the required multiple conformations would be plausible in terms of thermodynamic stability.

### 3.1. PrP^Sc^ Characterization and Strain Variation in Natural Hosts: CJD, FI, GSS, and VPSPr

**Figure 1:**
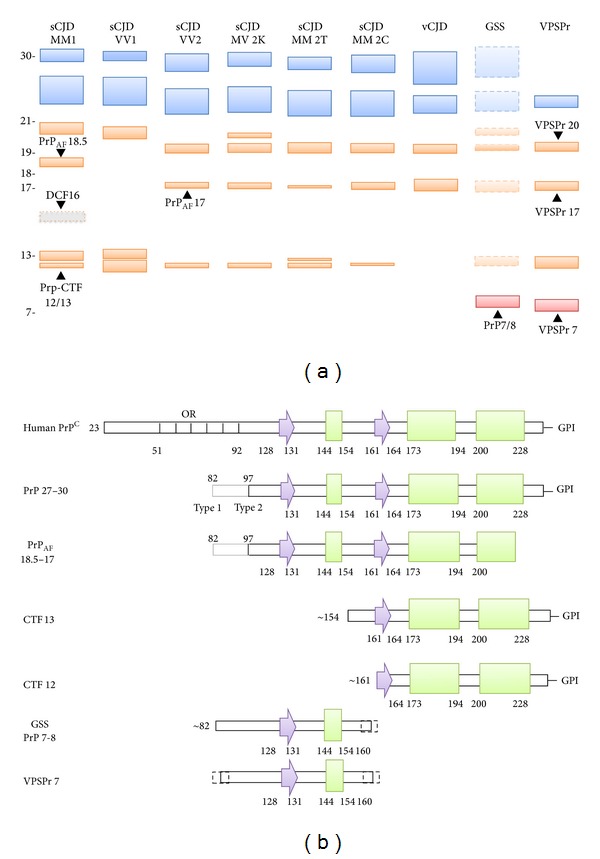
(a) Schematic representation of the spectrum of PrP^res^ fragments observed in human prion diseases and their electrophoretic profile. The unglycosylated forms of all PrP^res^ fragments with the glycosylation sites in their sequence are indicated in orange, while the fragments lacking these sites are shown in red. Among the glycosylated peptides, only the mono- and the diglycosylated forms of PrP^res^ 27–30 (18–21 kDa range) fragments are shown (in blue). The DCF16 fragment, which is generated only in partially denaturing conditions is labeled with a dotted line and a gray color. For GSS, the fragments that have been described only associated with specific *PRNP* mutations (e.g., P102L or A117V) are shown with dotted lines and in transparency. Molecular weights are indicated on the left in kDa. (b) Diagrams of the secondary structural elements of human PrP^C^ and of the PrP^res^ fragments observed in human prion diseases. Arrows are representative of *β*-strands and rectangles of *α*-helices and OR indicates the octapeptide repeats region. The secondary structure numbering has been derived from pdb (Protein Data Bank) id 2LSB (human PrP).

Five major clinicopathological phenotypes of human prion disease are currently recognized. These are CJD, FI, GSS, PrP-cerebral amyloid angiopathy, and VPSPr (phenotypic features of each form are reviewed in [15, 124–128]). The vast majority of human prion cases belong to CJD and occur in a sporadic fashion and worldwide. Only a small proportion of CJD cases are associated with *PRNP* mutations, in the form of familial or more properly genetic CJD (gCJD). Secondary CJD associated with inadvertent medical transmission is termed iatrogenic CJD (iCJD), and the only known zoonotic form of CJD, which is associated with exposure to BSE, is termed variant CJD (vCJD). VPSPr is a very recently described rare sporadic phenotype resembling GSS, FI can either occur sporadically or in a familiar form (FFI) associated with the D178N-129 M *PRNP* haplotype, while GSS and PrP-CAA phenotypes are tightly associated with mutations in the *PRNP* gene. In CJD, the prototype of human prion diseases, the characterization of PrP^Sc^ after PK treatment has led to the discovery of two major fragments of protease-resistant PrP^Sc^ (PrP^res^). The largest of these peptides, named type 1, has a relative electrophoretic mobility of 21 kDa and a primary PK cleavage site at residue 82 while the smallest, or type 2, has a relative molecular mass of 19 kDa and a primary cleavage site at residue 97 [114, 115, 129] (Figure 1). Based on the analysis of a large series of 300 sCJD cases it was shown that the two different PrP^res^ types can be associated with each of the three possible *PRNP* genotypes determined by the polymorphic codon 129 (methionine, M, or valine, V) and that the six different possible combinations between these two molecular variables significantly correlate with the clinico-pathological heterogeneity of sCJD [130]. Intriguingly, the two PrP^res^ types were also detected in the genetic and acquired forms of CJD, including vCJD, thus independently from the apparent etiology of the disease, that is, sporadic, inherited or acquired by infection [114, 129, 131], suggesting that the same prion strains are contributing to all forms of human TSEs. Furthermore, PrP^res^ types 1 and 2 were also found to cooccur in the same brain in about one-third of all sCJD cases [130, 132–134]. The results obtained in large series of cases indicate that the deposition of either type 1 or 2, when concurrent, is not random and is always characterized by the coexistence of phenotypic features previously described for the “pure” subtypes, a finding which strongly suggests that these cases harbour a mixture of prion strains.

The identification of an excess of pathological phenotypes (i.e., at least six) with respect to PrP^res^ types 1 and 2 dichotomy has prompted further attempts to identify PrP^res^ properties that would correlate with each disease phenotype. Using a standardized high buffer strength for brain homogenization, PK digestion at pH 6.9 with a high enzyme concentration, and long running gels, Notari et al. [137] showed that distinctive PrP^res^ properties can indeed be found in sCJD phenotypes sharing the same PrP^res^ type. For example, (i) PrP^res^ type 2 from MV cases shows a unique doublet band that differs from PrP^Sc^ type 2 in MM and VV cases, and (ii) type 1 PrP^res^ from VV cases migrates faster than type 1 PrP^res^ from MM1 and MV1 samples when PK digestion is performed at pH under 7.2 (Figure 1(a)).

A further fine tuning of the PrP^Sc^ signature associated with each CJD-associated strain has been obtained with the discovery that PrP^Sc^ aggregates include PrP^res^ C-terminal fragments with a relative mass of about 12 and 13 kDa (PrP-CTF12/13), in addition to PrP 27–30 (Figure 1). These fragments originate from the cleavage of PrP^Sc^ at residues 162–167 and 154–156 and vary in relative abundance among sCJD subtypes; in particular the peptide CTF-13 is present in significant amount in MM1 cases and is particularly abundant in VV1 subjects, whereas all PrP^res^ type 2-associated sCJD subtypes but the MM 2T, as well as vCJD, show only traces of this fragment [138]. Notari et al. [138] also identified a novel C-terminally truncated PrP^res^ fragment showing an apparent molecular mass of either ~18.5 kDa (when associated with type 1) or ~17 kDa (when associated with type 2). This fragment shares the primary N-terminal sequence with either type 1 or type 2 but lacks the very end of the C-terminus together with the GPI anchor (PrP_AF_ 18.5-17) (Figure 1). Finally, a fragment with an apparent molecular mass of about 16 kDa, which is only generated in partially denaturing conditions (DCF 16), has been detected in sCJD MM1/MV1 (Figure 1). Epitope mapping indicates that the fragment has an intact C-terminal end and is truncated in the region between residue 112 and residue 144. Taken together, these data suggest that each sCJD subtype can be associated with a specific profile of PrP^res^ fragments (PrP 27–30, PrP_AF_ 18.5-17, DCF 16, PrP-CTF12/13), possibly reflecting subtype-specific structural characteristics of the protein aggregate [138] (Figure 1).

PrP^Sc^ from different prion strains can also be typed through its glycoform ratio, that is the ratio between the three differently glycosylated isoforms of PrP^res^ 27–30 (i.e., diglycosylated, monoglycosylated, and unglycosylated) (Figure 1). In the large majority of CJD cases, PrP^res^ glycosylation is characterized by an overrepresentation of the monoglycosylated form [115, 130]. A rather grossly major distinction with diagnostic relevance has been introduced to distinguish the above-described “pattern A” from “pattern B” characterized by a predominance of the fully glycosylated form, the latter being found in vCJD [114, 116] or in gCJD and FFI linked to the E200K or D178N mutations, respectively [129, 139]. However, finer significant differences in PrP^res^ glycoform ratio have also been described among CJD subtypes with either “pattern A” or “pattern B” using either mono- or two-dimensional gel electrophoresis [130, 140].

Besides the strain typing approaches based on the analysis of the PrP^res^ fragments generated by PK cleavage and glycoform ratio, other approaches have focused on PrP^Sc^ detergent solubility and aggregate size, degree of protease-resistance, and conformational stability [141–143]. Kobayashi et al. [142] studied PrP^Sc^ aggregation in MM 1 and MM 2T sCJD (sFI) cases and found that the former has a larger aggregation size than that of the latter, a result which they also confirmed in case with the cooccurrence of PrP^Sc^ types 1 and 2. More recently, Saverioni et al. [111] have analyzed PrP^Sc^ protease resistance and aggregate size across the whole spectrum of human prions (all sCJD subtypes, sporadic FI (sFI), vCJD, and VPSPr) and found that the strain-specific PrP^Sc^ sensitivity varies over a 100-fold range of PK concentration and that these differences stem from both PrP^Sc^ aggregate stability and size.

Preliminary data on the conformational stability of PrP^Sc^ in CJD subtypes have also become available. Conformational stability assay (CSA), which measures the progressive loss of PrP^Sc^ PK-resistance after exposure to increasing concentration of GndHCl, showed that sCJDMM1 PrP^Sc^ is more stable than sCJDMM 2C PrP^Sc^ [143]. The same result was obtained with the conformation stability and solubility assay (CSSA), which measures the increase in solubility of PrP^Sc^ after exposure to increasing concentrations of GndHCl. [144]. Finally, both sCJDMM1 and VV2 PrP^Sc^ showed a higher stability than vCJD PrP^Sc^ in the conformation dependent immunoassay (CDI), which evaluates the increase in epitopes exposure after GndHCl denaturation [145].

According to Kim et al. [109] sPrP^Sc^ concentration and stability is in close correlation with the disease progression rate. This, in turn, would reflect the association between the strain-specific amount and stability of sPrP^Sc^ conformers and the efficiency in initiating the replication process *in vitro* [110].

Preliminary data obtained in three sCJD variants seem to suggest that both levels and stability of sPrP^Sc^ are good predictors of the progression rate in sCJD and that small oligomers of protease-sensitive conformers of PrP^Sc^ may govern conversion potency. In particular, when sPrP^Sc^ is less stable than rPrP^Sc^, as in sCJDMM1 and VV2, the difference in stability would correlate with less accumulated sPrP^Sc^ and a shorter duration of the disease, whereas when sPrP^Sc^ conformers are more stable than rPrP^Sc^, as in sCJD MM2, it would correlate with more accumulated sPrP^Sc^ and a longer disease duration [110]. sPrP^Sc^ oligomers, smaller in size than rPrP^Sc^ polymers, may be the most powerful in triggering *in vitro* amplification due to an increased surface availability for recruiting PrP^C^ molecules for conversion. So, the strain in which these sPrP^Sc^ conformers are most abundant would be the most efficient in amplification assays. In this regard, it is noteworthy that PMCA requires a sonication phase aiming to reduce the aggregation size of the seed. Although stimulating and sound with the current view of the biology of prions, the scenario depicted above must be taken with caution and definitely awaits confirmation by further investigations.

In addition to classical CJD variants and FI, human prion diseases include GSS and the recently described VPSPr. GSS is a familial disease which has been linked to missense, stop-codon, or insertional mutations in *PRNP*. The clinical phenotype in GSS is most commonly characterized by a progressive cerebellar syndrome, accompanied by extrapyramidal and pyramidal signs and cognitive decline, which may evolve into severe dementia [124]. However, a clinical variability, with either cognitive decline anticipating ataxia and rigidity or spastic paraplegia as a presenting symptom, has been observed. Neuropathological features associated with GSS disease vary substantially but always include PrP-positive multicentric amyloid plaques in the cerebellum and the cerebral cortex with or without associated spongiform change. Pioneering studies in GSS showed that purified amyloid preparations and the PrP^res^ obtained by *in vitro* proteolysis mainly comprise atypical unglycosylated 7-8 kDa PrP fragments with ragged N and C termini, primarily composed of mutant PrP, which are lacking in classic TSEs such as CJD and FI (Figure 1) [102, 146–151]. In keeping with the significant phenotypic heterogeneity of the disease, however, it was also shown that the western blot profile of PrP^res^ in GSS may comprise additional PrP^res^ fragments of higher molecular weight, including the CJD-associated PrP^res^ type 1 (Figure 1) [102, 146]. More specifically, GSS affected subjects carrying the most common GSS mutation (P102L) may either show a rapidly progressive CJD-like phenotype with both spongiform changes and amyloid plaques correlating with the cooccurrence PrP^Sc^ type 1 and the 8 kDa fragments or show a more slowly progressive “pure” GSS phenotype correlating with the presence of amyloid plaques and the 8 kDa PrP fragment [102, 146]. Finally, GSS associated PrP^Sc^ has also been reported to be unusually protease sensitive, at least in a subgroup of cases [102, 105, 106, 152]. Interestingly, when compared with CJD PrP^Sc^, this increased proteolytic sensitivity of PrP^Sc^ does not correlate with a distinct aggregate sedimentation profile, suggesting that it is not due to a lower size of aggregates but rather to differences in their conformation [105, 106].

VPSPr is a recently described atypical variant of sporadic human prion disease, clinically characterized by language deficits, cognitive impairment, motor signs, especially Parkinsonism and ataxia, and an average longer clinical course than CJD [104, 153–156]. The disease can apparently affect all 3 codon 129 genotypes, although this genetic variability affects both susceptibility and phenotypic expression [104]. Pathologically, VPSPr is characterized by the spongiform change, which is especially seen in neocortical and subcortical regions of the cerebrum, such as the striatum and thalamus, and PrP-positive amyloid microplaques in the cerebellar molecular layers [104, 153].

Despite the clear differences in the clinicopathological phenotype between VPSPr and GSS, the characterization of PrP^Sc^ physicochemical properties has highlighted strong similarities which have led to the hypothesis that the former may represent the sporadic variant of the latter [153]. Indeed, PrP^res^ in VPSPr shows a striking, ladder-like, electrophoretic profile comprising at least 4 bands, including a prominent one migrating at about 8 kDa (Figure 1). Furthermore, the abnormal PrP shows a variable degree of PK-resistance according to the codon 129 genotype; it is highly protease-sensitive in subjects with VV, whereas it shows a degree of resistance comparable to some sCJD types in subjects MV or MM at codon 129 [104, 111]. A very recent study also demonstrated that VPSPr shares PrP^Sc^ features with a known familial CJD linked to a valine to isoleucine mutation at residue 180 of PrP (fCJDV180I), exhibiting similar patterns of glycosylation and protease cleavage [157].

### 3.2. Transmission Studies with Human Prions

The first characterization of the transmissible, strain-related properties of human sporadic prion isolates was accomplished in transgenic mice. Inocula from a single sFI (i.e., MM 2T) case produced disease characteristics that differed from those induced by sCJD MM1 as well as from genetic CJD cases carrying the E200K-129M or the V210I-129M haplotypes [158]. Preliminary data concerning the transmission properties of other sCJD subtypes became available a few years later [159, 160], but only recently the reevaluation of the National Institutes of Health series of prion disease transmitted to non-human primates [131, 161] and more comprehensive experimental transmissions to transgenic mice [153, 154] have substantially clarified the issue of the extent of strain variation in sporadic human prion disease and provided answers to the crucial question of how the current classification relates to different strains of sCJD. The results of these studies indicate that, besides the MM 2T variant already mentioned above, four out of five of the other neuropathologic and molecular “pure” types of sCJD defined by the classification of Parchi et al. [130, 133] behave indeed as different strains of agent. Most importantly, sCJD MM1 and MV1 isolates have identical transmission properties, which significantly differ from those of sCJD VV2 or MV 2K. Furthermore, both the sCJD MM 2C and sCJD VV1 subtypes behave differently from each other and from the other isolates after transmission [162]. However, at variance with the sCJD MM1/MV1 and VV2/MV 2K strains, only single cases of sCJD MM 2C, MM 2T, and VV1 have been examined, with the assumption that transmission characteristics of a single case will be representative of the particular subgroup. Thus, the results obtained for these rare subtypes, although clear and somehow expected, await confirmation [131, 162, 163]. Familial and acquired forms (except for vCJD; see below) are likely linked to the same pool of strains isolated from sCJD. For example, inocula from carriers of E200K and V210I mutations affected by the MM1 CJD phenotype showed the same transmission properties of sCJD MM1 inocula when propagated in Tg mice, non-human primates, or bank voles [92, 131, 160]; similarly, experimentally transmitted kuru reproduced the same clinico-pathological and biochemical features of VV2 and MV 2K sCJD [131]. Finally, similar properties have been observed by FFI and sFI prions when propagated into Tg mice [34, 158]. In contrast to prions propagated in classical CJD and kuru, the transmission properties of vCJD prions are strikingly distinct and have established vCJD as a distinct human prion strain [164, 165]. The vCJD prions transmit disease to wild-type mice far more efficiently than any other form of human prion disease [164–166] and in transgenic mice faithful propagation of the vCJD phenotype is dependent upon homozygous expression of human PrP 129 methionine [165, 167–170]. Transgenic mice homozygous for human PrP 129 valine show a pronounced transmission barrier to vCJD prions and propagate a distinct clinical-pathological phenotype [165, 167–169, 171]. As a consequence, the possibility that the BSE-vCJD strain may be associated with other human pathological phenotypes besides that observed in subjects carrying MM at codon 129 should not be dismissed.

With the significant exception of the GSS P102L associated with spongiform changes and PrP^Sc^ type 1, which shows CJD-like transmission properties, GSS variants have been more difficult to transmit to animals than CJD or FFI [161, 172]. This has led to the suggestion these GSS phenotypes are not true prion diseases (e.g., TSEs) and are better designated as nontransmissible proteinopathies. In more recent studies, however, the use of transgenic mice carrying GSS mutations such as A117V or the mouse equivalent of P102L has led to the finding that brain tissue from GSS patients carrying the corresponding mutation could induce a pathological phenotype into these mice, although with some significant differences between the two models [173, 174]. More specifically, in the first, the inoculation of brain extracts from a GSSP102L patient with no spongiform change caused almost no clinical disease but induced striking PrP-amyloid deposition in brains of several recipient mice; extracts of those brains failed to transmit neurological disease on further passage but again induced PrP-amyloid plaques in recipient mice [173]. In the second study, instead, the transmission of a more typical TSE phenotype, including the deposition of classic protease-resistant PrP^Sc^ 27–30, has been obtained in 117VV HuPrP transgenic mice challenged with A117V prion isolates [174]. Thus, especially according to this latter result, GSS may also be considered a true prion disease, although much less prone than CJD to transmit, possibly because it is characterized by the formation of less stable PrP^Sc^ aggregates.

### 3.3. PrP^Sc^ Properties and Strain Variation in Natural Hosts: Scrapie, BSE, and CWD

#### 3.3.1. Scrapie

Biochemical typing of natural scrapie isolates has been largely based on the assessment of PrP^res^ electrophoretic mobility, glycoform ratio, and epitope mapping of PK-cleavage sites using different monoclonal antibodies. The use of other approaches such the analyses of PrP^res^ protease-resistance and conformational stability of PrP^res^ has been, so far, limited.

**Figure 2:**
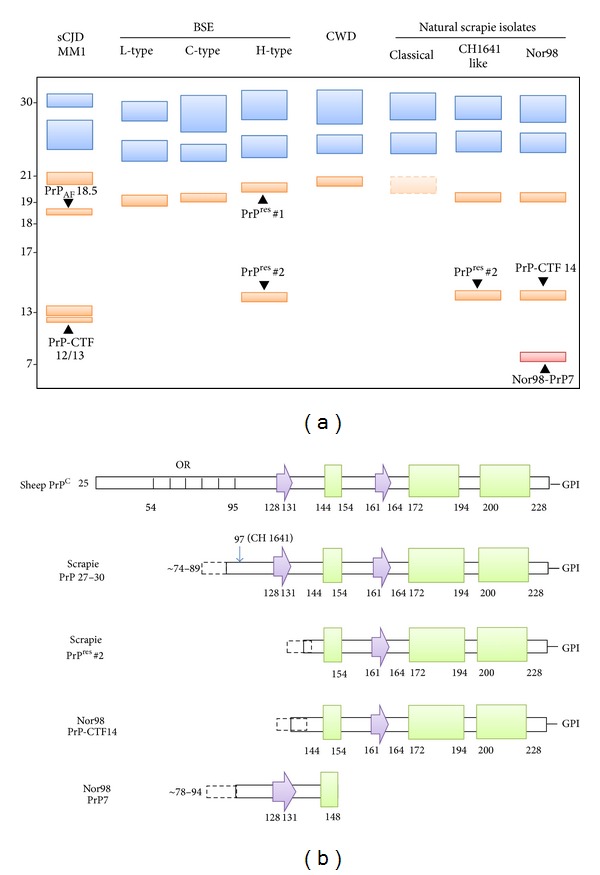
(a) Schematic representation of the spectrum of PrP^res^ fragments observed in animal prion diseases and their electrophoretic profile. The unglycosylated forms of all PrP^res^ fragments with the glycosylation sites in their sequence are indicated in orange, while the fragments lacking these sites are shown in red. Among the glycosylated peptides, only the mono- and the diglycosylated forms of PrP^res^ 27–30 (18–21 kDa range) fragments are shown (in blue). To facilitate the comparison with human forms, the profile of MM1 sCJD associated PrP^res^ is shown; note that the unglycosylated band of sCJDMM1 PrP^res^ has the same electrophoretic mobility of that of CWD as reported by Xie et al. [14]. (b) Diagrams of the secondary structural elements of sheep PrP^C^ and of the PK-resistant PrP fragments observed in classical and atypical Nor98 scrapie. Arrows are representative of *β*-strands and rectangles of *α*-helices and OR indicates the octapeptide repeats region. The secondary structure numbering has been derived from pdb (Protein Data Bank) id 1XYU (sheep PrP).

Despite the known diversity of classical scrapie strains that have been isolated in wild-type mice [83] or hamster [175, 176], the identification of strain-specifc PrP^res^ signatures in sheep with natural scrapie has proved to be challenging [177–182]. Indeed, the molecular signature of most isolates of classical scrapie comprises an unglycosylated PrP^res^ with a “high” (h-type) molecular mass (i.e., in the range of human PrP^res^ type 1 and including the epitope recognized by the N-terminal P4 antibody), whereas only a few cases show a PrP^res^ profile with a “low” (l-type) electrophoretic mobility (i.e., in the range of human PrP^res^ type 2 and not labeled by P4), similar to that seen in BSE or experimental scrapie strain CH1641 (Figure 2). Similarly, PrP^res^ glycoform ratios did not clearly differ from those found in cattle-BSE and did not reveal distinct subgroups of classical scrapie [180–185] (Figure 2). Some evidence for a strain-related heterogeneity of PrP^Sc^ associated with classical natural scrapie isolates derives from CDI analysis. It has been shown that PrP^Sc^ extracted from sheep with the VRQ/VRQ *PRNP* genotype has higher levels of PK-sensitive PrP^Sc^ than the PrP^Sc^ associated with ARQ/ARQ [108]. Furthermore, the two isolates propagated in mice are associated with two PrP^Sc^ with distinct conformational stability, with the PrP^Sc^-VRQ inocula being more sensitive to denaturation than the other [186].

The unusual scrapie isolates with a l-type PrP^res^ profile, designated as CH1641-like, have for some time posed a diagnostic challenge because of the similarities with the PrP molecular properties of experimentally transmitted BSE to sheep. Immunoblot assays have shown that they share a migration pattern similar to the unglycosylated PrP^res^ fragment but have different levels of diglycosylated PrP^res^ [178] (Figure 2). More recently, however, it has been found that the PrP^res^ associated with the CH1641-like isolate clearly differs from BSE-PrP^res^ by the presence of an additional band at approximately 14 kDa, which is specifically recognized by the C-terminal antibody SAF84 (Figure 2) [187]. This additional PrP^res^ fragment was also observed after transmission in a transgenic mouse model (TgOvPrP4) of both the natural CH1641-like isolate [188, 189] and the CH1641 experimental scrapie isolate that was originally isolated from a British scrapie case and maintained by serial transmissions in sheep [190]. Unlike CH1641 this PrP^res^ fragment was not detected in the scrapie strains with h-type PrP^res^ (C506M3, Chandler, and 79A), arguing that PrP^res^ 14 kDa preferentially associates with l-type PrP^res^ [189]. Intriguingly, both l-type and h-type PrP^res^ were detected in the brain of TgOvPrP4 infected with some scrapie isolates [187], which strongly suggests that the two phenotypes found in mice could be the result of the cooccurrence of two strains in these sheep. Indeed the possible existence of a mixture of strains from a single scrapie case, which can only be separated by biological cloning, has been documented following bioassay in mice or hamsters [191, 192].

In 2003 an atypical scrapie strain (Nor98) was described in five sheep from Norway [193]. Scrapie cases similar to Nor98 were later detected in other European countries [194–196] and in the United States [197]. Western blotting analysis of Nor98-affected brain extracts has allowed the identification of a peculiar PrP^res^ electrophoretic profile consisting of multiple protein bands including a prominent band of relatively low molecular mass that was initially reported to migrate around 12 kDa [186].

In particular, the use of different mAbs raised against epitopes located in the middle and in the C-terminal regions of PrP has allowed the identification of two previously unrecognized fragments, respectively, designated as Nor98-PrP7 and PrP-CTF14 (Figure 2). Nor98-PrP7 is a PK resistant N- and C-terminally truncated fragment with a molecular weight of 7 kDa which is not affected by PNGase F treatment, while PrP-CTF14 is a C-terminal fragment migrating at 14 kDa after deglycosylation. Interestingly, both fragments showed an increased protease sensitivity when compared to PrP^Sc^ in classical scrapie, suggesting that the PrP^Sc^ associated with the two diseases have a different conformation [198].

The intracerebral inoculation of a panel of atypical/Nor98 scrapie isolates into mice overexpressing the ovine prion protein (Tg338) suggests that a single prion strain is responsible for atypical scrapie [199]. Using a set of PrP-specific monoclonal antibodies two distinct C- and N-terminally ragged PK-resistant PrP^res^ fragments of approximately 8 kDa and 5 kDa which are differently truncated at their C-termini were detected, thus confirming the complexity and the specificity of the molecular PrP^res^ phenotype of these atypical scrapie isolates [199] and its similarities with some human TSE variants such as GSS-P102L and VPSPr (Figures 1 and 2) [200].

#### 3.3.2. Bovine Spongiform Encephalopathy (BSE)

On the basis of the electrophoretic profiles of the unglycosylated band of PrP^res^, three different BSE phenotypes are currently recognized: the classical BSE (C-type) and two atypical BSE variants showing, respectively, a lower (L-type) and a higher (H-Type) relative molecular mass of PrP^res^ in comparison to the C-type [113, 181, 201, 202] (Figure 2(a)).

Early evidence suggested that BSE was caused by a prion strain characterized by an efficient ability to overcome the species barrier and with a PrP^res^ signature featuring a lower relative molecular mass compared to the PrP^res^ associated with classic scrapie (and CWD) and a marked predominance of the high molecular weight glycoform [164, 203].

In 2004, however, a distinct phenotype of bovine amyloidotic spongiform encephalopathy (BASE or L-type) [113], correlating with a PrP^res^ showing a slightly lower electrophoretic mobility than the PrP^res^ of the C-type and a predominant monoglycosylated isoform, was found [201]. The evidence that BSE and BASE are caused by two distinct prion strains is supported by transmission experiments showing that the inoculation of BSE or BASE brain homogenates in transgenic mice (Tgbov XV) causes two distinct phenotypes [204]. Noteworthy, BASE was also shown to convert into the classical BSE strain upon serial transmission to inbred mouse lines, which has raised the hypothesis that BSE originated from BASE [205].

Intriguingly also the H-type BSE, first described by Biacabe et al. in 2004 [201], can recapitulate most of the phenotypic features of classical BSE after cross-species transmission experiments in wild-type mice [206]. Compared with the C-type, the H-type strain is characterized by an extended N-terminus of PrP^res^ and by the presence of two distinct PrP^res^ cleavage products, PrP^res#1^ (19–30 kDa), showing a slightly higher electrophoretic mobility than the PrP^res^ of the C-type, and PrP^res#2^ (14–24 kDa), characterized by a more C-terminal cleavage [207] (Figure 2(a)). This typical H-type PrP^res^ banding pattern was also described in a BSE case associated with a *PRNP* mutation (E211K) [208].

#### 3.3.3. Chronic Wasting Disease (CWD)

CWD, like scrapie, is a prion disease mainly transmitted via an environmental route [209]. Although the horizontal transmission of CWD among cervids by direct or indirect contacts is remarkably efficient, its transmission to different species has yet to be fully clarified [210–212].

The PrP^res^ electrophoretic profiles of CWD-affected animals and of sCJDMM1 have led to the observations that they share some similarities as shown by the conformational stability assay and by the observation that in both samples the unglycosylated PK-resistant isoform migrates at 21 kDa, thus indicating a similar conformation of the PK resistant cores. However, the two PrP^res^ do not display a similar glycoform profile with a prevalence of the diglycosylated isoform in the CWD PrP^res^, as observed in BSE and in vCJD [14] (Figure 2(a)). The same electrophoretic and glycoform profiles were also observed in two different CWD strains (CWD1 and CWD2) which were identified after the inoculation of different CWD isolates in Tg mice expressing cervid PrP (Tg (CerPrP)1536^+/−^) [213].

Interestingly the PrP^res^ immunoblot analysis of white-tailed deers orally inoculated with the CWD agent revealed that in Q95H/G96S animals the unglycosylated fragment migrates at lower molecular weight and the level of PK-resistance seems to be reduced, suggesting the generation of a PrP^res^ with different properties which the PrP^res^ generated in the other infected cervids [214].

## 4. Role of **PrP^Sc^** in Prion Toxicity and Neurodegeneration

The understanding of the mechanisms of toxicity resulting from misfolding and ordered aggregation of proteins involved in prion disease and many others neurodegenerative diseases remains an open question and a research priority. Indeed, in none of these diseases are the mechanisms of toxicity completely clear. While a large body of evidence indicates that the misfolded protein aggregates are the cause of the neurodegeneration, many studies link this toxicity to the existence of various intermediate structures, likely in the oligomeric state, prior to the fiber formation and/or their specific interaction with membranes [215, 216]. Indeed, in prion diseases it is well established that, in the absence of GPI-linked PrP^C^, PrP^Sc^ is innocuous, suggesting that PrP oligomers and fibrils are not toxic *per se* [217], and that PrP^C^ may act as mediator of the toxic signal. Furthermore, the importance of certain physicochemical properties of the protein fragments forming the aggregate, such as size and glycosylation state, has also been highlighted by studies in prion disease, which uniquely comprise a wide range of disease phenotypes allowing for extensive molecular and clinicopathological correlations [125].

### 4.1. Insights from Studies on Naturally Occurring and Experimentally Transmitted Prion Diseases

From the study of affected brains we have learned that the events that are triggered by prion neuroinvasion and that result in neurodegeneration may vary significantly both in terms of resulting histopathology and speed of the neurodegenerative process. In humans the clinical course of a prion disease may range from a few weeks to at least one decade, and evidence from experimental transmissions and acquired prion diseases indicate that a similar heterogeneity likely characterize also the preclinical phase.

Histopathologically, while most prion diseases, including CJD, BSE, CWD, and most of scrapie cases (i.e., the classic transmissible spongiform encephalopathies or TSEs), are characterized by the triad of spongiform change, gliosis, and neuronal loss, some rare but very informative variants such as FI, GSS, or PrP-CAA may show very subtle or even absent spongiform change or be characterized by prominent extracellular amyloid plaques accumulating either in the neuropil or around blood vessels. Most significantly, in contrast to CJD, in which the abnormal PrP^Sc^ aggregates mainly consist of full-length protein together with GPI-anchored, N-terminal fragments truncated between residue 82 and residue 104 [129], in GSS or PrP-CAA affected patients the abnormal PrP plaque amyloid that accumulates is composed primarily of truncated internal PrP fragments (e.g., residues 82–153) that lack the GPI anchor and the glycosylated moiety [102, 146, 152]. In this respect, GSS patients carrying the P102L mutation can be considered a “quasinatural” experimental model. Indeed, while in some of these patients pure GSS histopathological features correlate with the presence of the GPI-anchorless PrP fragment, in others mixed CJD/GSS features (e.g., widespread spongiform changes cooccurring with amyloid plaques) correlate with the deposition of both types of PrP^Sc^ forms (e.g., GPI-anchored and glycosylated N-terminal PrP^Sc^ fragment + truncated internal PrP fragments lacking the GPI anchor). These observations strongly support the idea that PrP fragments have different neurotoxicities and cause distinct lesions as a consequence of their different properties, such as aggregation propensity [102]. In particular, the longer duration of illness in GSS patients can be explained postulating that the short GPI-anchorless PrP^res^ fragments have a higher tendency toward aggregation and plaque formation and thus provide a relative protection with less neuronal dysfunction than the 21- or 19-kDa PrP^res^ glycosylated fragments or full length PrP^Sc^ associated with CJD that form more diffuse and smaller deposits. Consistent with this hypothesis is also the observation that, among the GSS P102L patients, those showing the mixed CJD/GSS phenotype have, on average, a significantly shorter course [102].

More recently, the evidence obtained from studies on CJD and GSS patients has received strong support from a transgenic mouse model expressing anchorless PrP [218]. In these Tg44 mice scrapie infection results in an unusual type of slow fatal prion brain disease distinguished by widespread deposition of PrP^Sc^ amyloid in the CNS [219] and in extraneural sites such as heart, brown fat, white fat, and colon [220, 221]. In the CNS of infected Tg44 mice the gray matter vacuolation typical of prion diseases is minimal, and PrP^Sc^ is primarily deposited as perivascular amyloid [219]. In this model, most of the typical clinical and neuropathological characteristics of scrapie are either absent or greatly reduced, despite the accumulation of brain PrP^Sc^ to levels comparable to those in scrapie-infected wild-type mice. This reduced brain damage could be due either to a need for anchored PrP^C^ on brain cells for toxicity induced by PrP^Sc^ and/or to a lower pathogenicity of PrP^Sc^ amyloid plaques compared to the more dispersed, amorphous, and membrane-associated PrP^Sc^ deposits seen in most other prion diseases. These findings highlight the role of GPI anchor in TSE pathogenesis [222]. It is likely that the anchoring of PrP^Sc^ aggregates to membranes by the GPIs could distort its local structure, composition, flexibility, fluidity, dynamics, integrity, and, hence, functionality. The results of several elegant EM studies corroborate these observations by showing that in all the naturally occurring TSEs of animals, as well as in experimental scrapie models of mice, there are a number of distinctive membrane changes, including membrane microfolding, membrane clefts, and abnormal endocytosis of dendrites, which are both directly linked to PrP^Sc^ and appear to be unique to prion diseases [223, 224]. These changes, however, were absent from Tg mice expressing only anchorless PrP and other Tg mice developing large amyloid plaques composed of abnormal prion protein [225].

While a definite progress has been made in understanding the divergent molecular pathology between classic TSEs and the “anchorless” PrP-amyloidosis, much less is known about the molecular basis of the different “neurotoxicity” associated with the various prion strains. Indeed, differences in the molecular and cellular pathology that correlate with the severity of the clinical phenotype have also been observed among classic TSEs such as sCJD. Subjects affected by the most common sCJD variant (e.g., the MM1 subtype), for example, do not accumulate higher amounts of PrP^Sc^ or develop more severe histopathological changes than the other sCJD variants despite their very rapid clinical course, sometimes lasting less than a month [115, 226]. Similarly, in a recent study in which we have correlated the amount of PrP^Sc^ deposition with the extent of microglial activation across the whole spectrum of sCJD subtypes, including the MM 2T or FI, we found that the degree of microglial activation differs significantly between disease subtypes and, above all, it does not correlate with the overall amount of PrP^Sc^ accumulation (Strammiello R and Parchi P, unpublished). Intriguingly, the most significant difference in the ratio between PrP^Sc^ amount and HLA-DR load was seen between two subtypes, the MM 2C and the MM 2T, sharing the average disease duration, codon 129 MM genotype, and PrP^Sc^ type 2. Overall, these data add to previous observations indicating that many critical properties of prions, including neurotoxicity, appear unrelated to the overall amount of PrP^Sc^ deposition. Furthermore, they indicate that there are strain-related differences in the apparent “neurotoxicity” associated with PrP^Sc^ deposition that must be addressed.

Another intriguing and largely unexplained issue of prion pathology concerns the regional specificity. In this respect, the study of FI, which is by far the most peculiar disease phenotype among those characterized by a “classic” PrP^Sc^ 27–30 deposition, has been very informative. The histopathological hallmark of FI, especially of the familial form linked to the D178N-129M *PRNP* haplotype, is a severe neuronal loss in the medial thalamic and inferior olivary nuclei [126]. These changes develop early since they are found in all affected subjects, irrespectively of the disease duration; furthermore they are found associated with amounts of PrP^Sc^, which are at least tenfold lower than those detected in other sCJD subtypes where the neuronal loss in the thalamus is rarely so severe. In contrast, in the neocortex and, to a lesser extent, in the limbic cortex and the striatum of FFI patients, the amount of PrP^Sc^ increases with the duration of symptoms and eventually accumulates in significantly higher amounts than in the thalamus [227, 228]. Furthermore, the higher extent of PrP^Sc^ deposition correlates with the appearance of spongiform changes rather than with the degree of neuronal loss which remains milder than in the thalamus.

In conclusion, significant differences in the “neurotoxicity” associated with PrP^Sc^ deposition are also seen among classic TSE subtypes. However, in contrast with GSS, no significant data have been collected to explain how PrP^Sc^ may mediate these heterogeneous effects.

### 4.2. Insights from Studies on Animal Models

The first studies documenting the progression of neurodegeneration in prion disease dates back half a century and preceded the discovery of the prion protein. At that time experimental transmissions in primate and murine models already established that the appearance of spongiform change precedes neuronal loss and reactive astrogliosis [229]. It was later found that PrP^Sc^ deposition almost invariably represents the earliest event of the pathological cascade, which is immediately followed by microglial activation and the appearance of spongiform change. It was also found that the conversion of PrP^C^ into PrP^Sc^ is critical to the neurotoxicity associated with prion diseases since neither loss of PrP^C^ function nor deposition of PrP^Sc^ in absence of PrP^C^ expression is sufficient to cause the prion-associated pathology [230, 231]. Having established the central role of both PrP^Sc^ and PrP^C^ in prion pathogenesis, the critical issue has progressively become the search for a link between PrP^Sc^, neurotoxicity, and infectivity. Although the temporal and anatomical correlation between PrP^Sc^ formation and the development of infectivity and neuropathological changes is often obvious in prion disease, the overall correlation between PrP^Sc^ levels, infectivity, and neurotoxicity can be weak or even absent. For example, transgenic mice expressing some mutant forms of PrP^C^ that lack certain domains spontaneously develop neurological disorders, but no infectivity and bona-fide PrP^Sc^ are associated with prion protein aggregates accumulated in brain tissue of these animals [27, 232]. On the other hand, mice expressing GPI-anchorless prion protein show high levels of infectious PrP aggregate deposits, but reduced neurodegeneration compared to prion-infected wild-type mice [218]. Finally, there are subclinical infections in which there is abundant PrP^Sc^ but little symptomatology, for example, after inoculation of hamster prions into mice [233, 234]. Thus, it appears that infectious and neurotoxic forms of PrP could represent distinct molecular species, a view which is also supported by a recent study showing that prion propagation in brain proceeds via two distinct phases. More specifically, it has been shown that a clinically silent exponential phase, which rapidly reaches a maximal prion titre and is independent by PrP^C^ expression, is followed by a plateau phase, which determines time to clinical onset in a manner inversely proportional to prion protein concentration [235]. Notably, however, the same data would also fit the model of PrP^C^-mediated PrP^Sc^ toxicity (see below), without requiring the existence of a toxic PrP as a distinct entity [236].

Despite this largely unsolved complexity, as for other protein aggregation diseases, PrP^Sc^ oligomers currently attract most of attention and appear to be the preferred researcher's candidate to explain both prion toxicity and infectivity. However, while there appears to be little doubt that infectious prion particles consist of small PrP oligomers, it is much less clear whether oligomers, and if so which oligomers, are involved in prion toxicity. As far as the mechanism of mediated toxicity is concerned, current evidence supports the view that small oligomers formed on membrane-bound GPI-PrP^C^ may act by compromising the integrity of cellular membranes or, more likely, by mediating a neurotoxic signal triggered from the extracellular milieu by PrP^Sc^. Alternatively, PrP^C^ may disrupt the endosomal compartment after being internalized [237]. Lines of evidence suggesting that PrP^Sc^ neurotoxicity may involve impairment of the normal physiological activity of PrP^C^ have also been gathered, especially from the study of mutant forms of PrP that produce spontaneous neurodegeneration in transgenic mice without the formation of infectious PrP^Sc^ (reviewed in [238]). For example, Tg (PrPΔ32–134) mice, which express an N-terminally truncated form of PrP, spontaneously develop a neurodegenerative phenotype that is stoichiometrically reversed by coexpression of wild-type PrP, but only partially rescued by coexpression of a PrP^C^ isoform carrying an insert mutation. The rescuing effect of wild-type PrP would implicate a molecular target for PrP, which is presumably a receptor or another cell-surface complex capable of transducing a signal to the interior of the cell. Based on these evidences, Harris and collaborators [239] have proposed that PrP^Sc^ (or other toxic forms of PrP), by interacting with the same putative membrane target, may subvert a normal function of PrP^C^ to generate a neurotoxic signal. Although of significant interest, the proposed mechanism is in apparent contrast with the dominant mode of inheritance of familial prion diseases. Furthermore, the connection between the neurotoxic mechanisms activated by artificial mutants and those operative in “natural” prion diseases of humans and animals remain to be demonstrated. Whatever the nature and the mechanism of action of the toxic molecular species, there is a growing body of data to show that it is the synapses that are the first or most susceptible component of the neuron to succumb in the disease process rather than the death of the cell soma. Compromised synaptic function is currently thought to underlie the earliest symptoms in several neurodegenerative diseases, and loss of synapses, spines, and dendrites is thought to precede the loss of neuronal cell bodies [240–243]. Using an engineered mouse model Mallucci and collaborators [244] have shown that the block of PrP^Sc^ formation by knocking out PrP^C^ in prion-infected mice during the course of disease prevented neuronal loss and progression to clinical disease. PrP knockout produced both long-term survival and neuroprotection and the disappearance of early spongiform change, thus indicating that spongiosis is a predegenerative change occurring in neurons which may represent an early morphological marker of functional impairment [244].

Using the same model, this group of researchers has recently demonstrated that the decline of synapse number and transmission is associated with an abrupt loss of synaptic proteins [245]. PrP replication and the consequent rise of PrP levels during disease would cause a sustained induction of the cellular unfolded protein response (UPR). Rising levels of unfolded proteins in the ER would cause the phosphorylation of PERK-P, followed by that of eIF2a, which ultimately causes a reduction of new protein synthesis. The resulting chronic blockade of protein synthesis would lead to synaptic failure, spongiform changes, and, ultimately, neuronal loss. Based on these findings, it has been proposed that the key trigger to prion neurodegeneration is the continued, unchecked activation of the UPR due to the rising levels of PrP during disease, with fatal repression of translation rates.

### 4.3. The Role of Microglia in PrP^Sc^ Clearing and Prion Disease-Associated Neurodegeneration

A major theme in studies of the role of microglia in neuropathology is the dichotomy between their contributions to neurodegeneration *versus* neuroprotection. Prion diseases are not an exception to this theme. Lines of evidence indicate that PrP^Sc^ can be efficiently cleared from the brain and that phagocytosis by microglia represents a prominent clearing mechanism [246, 247]. On the other hand, it has also been shown that activated microglia may assume an aggressive phenotype and release inflammatory cytokine fostering neuronal apoptosis and neurodegeneration [248].

Recent studies have contributed to shed some light into the molecular events regulating microglial activation during prion infection. In murine prion disease, the microglia was shown to activate early in the disease process, even in the absence of widespread histologically detectable PrP^Sc^ deposits [249]. This activated phenotype, which has been referred to as anti-inflammatory or benign, shows low levels of inflammatory cytokines and readily detectable levels of TGF-*β* and PGE2 [241, 250]. While there is no evidence that the enhanced levels of PGE2 are detrimental, nor that TGF-*β* is injurious, this situation may significantly worsen in the presence of systemic inflammation. Indeed, when mice were challenged systemically with endotoxin to mimic an intercurrent infection, this maneuver led to a dramatic switch in the microglial phenotype with an aggressive inflammatory cytokine profile and increased neuronal apoptosis [248]. This concept of rapid switching of the microglia phenotype is of course entirely in keeping with what is known about the degree of plasticity of the cells of the macrophage lineage. Systemic inflammation has a profound impact on a number of other animal models of neurological disease [251] and accelerates cognitive decline in Alzheimer's patients [252].

## Figures and Tables

**Table 1:** Cofactors enhancing PrP^C^ conversion *in vitro*.

Cofactor	Experimental setting		Results	Refs.
Pentosan polysulfate (PPS)	Cell-free conversion assay	Hamster and mouse [^35^S] GPI(-) PrP^C^ seeded with brain derived PrP^res^ from infected hamsters (263 K) and mice (87 V)	(i) PPS increases the rate of formation and the yield of [^35^S] PrP^res^ (ii) PPS facilitates conversion of both Mo and SHa [^35^S] GPI(-) PrP^C ^at different temperatures	[44]

Heparin	Cell-PMCA	Cell lysates plus exogenously expressed HuPrP seeded with sCJD, vCJD, and hamster-adapted scrapie 263 K	(i) Both low and high molecular weight heparin enhance PMCA efficiency (ii) Seed-dependent effect of heparin on amplification efficiency	[45]

Sulfated dextran compounds	PMCA	PrP^Sc^ derived from BSE-infected cattle brain diluted in PrP^C^ substrate	(i) Enhanced BSE PrP^Sc^ amplification (ii) Amplified PrP^Sc^ induce lesions typical of prion disease in TgBoPrP	[135]

Synthetic poly (A) RNA	PMCA	Normal and diluted scrapie brain homogenate	(i) Stochastic* de novo* formation of PrP^Sc^ molecules from unseeded purified substrates (ii) Both amplified Sc237 or 139H PrP^Sc^ and *de novo* PrP^Sc^ molecules cause scrapie in inoculated Syrian hamsters	[136]

Phosphatidylethanolamine (PE)	PMCA	recPrP substrate with a recPrP^Sc^ seed	(i) Generation of infectious prions (ii) PE supports prion propagation using PrP molecules from multiple animal species	[55]

RNA from normal mouse liver plus POPG	PMCA	Normal mouse brain homogenate seeded with recPrP	(i) *In vitro* generated recPrP^res^ (ii) recPrP^res^ propagates its PK-resistant conformation to endogenous PrP^C^ (iii) recPrP^res^ causes *bona fide* prion disease in wild-type mice	[50]
